# Phosphate Adsorption by Silver Nanoparticles-Loaded Activated Carbon derived from Tea Residue

**DOI:** 10.1038/s41598-020-60542-0

**Published:** 2020-02-27

**Authors:** Van Tuyen Trinh, Thi Minh Phuong Nguyen, Huu Tap Van, Le Phuong Hoang, Tien Vinh Nguyen, L. T. Ha, Xuan Hoa Vu, T. T. Pham, Thi Nu Nguyen, N. V. Quang, X. C. Nguyen

**Affiliations:** 1 Institute of Environmental Technology, Vietnam Academy of Science and Technology, 18 Hoang Quoc Viet road, Ha Noi city, Vietnam; 2 Faculty of Environment and Chemical Engineering, Duy Tan University (DTU), 254 Nguyen Van Linh road, Da Nang, Vietnam; 3 Faculty of Natural Resources and Environment, TNU-University of Sciences (TNUS), Tan Thinh Ward, Thai Nguyen City, Vietnam; 4 Faculty of Civil and Environmental Engineering, Thai Nguyen University of Technology (TNUT), Tich Luong Ward, Thai Nguyen City, Vietnam; 5 Faculty of Engineering and IT, University of Technology Sydney (UTS), Box 123, Broadway Sydney, PO Australia; 6 Faculty of Physics and Technology, TNU-University of Sciences (TNUS), Tan Thinh Ward, Thai Nguyen City, Vietnam; 7 Institute of Research and Development, Duy Tan University, Da Nang, 550000 Vietnam; 8 Cao Bang Teacher’s Training College, De Tham road, Cao Bang town, Cao Bang Province, Vietnam; 9 Faculty of Chemistry, Ha Noi Pedagogical University 2, Vinh Phuc, Vietnam; 10 Laboratory of Advanced Materials Chemistry, Advanced Institute of Materials Science, Ton Duc Thang University, Ho Chi Minh City, Vietnam; 11 Faculty of Applied Sciences, Ton Duc Thang University, Ho Chi Minh City, Vietnam

**Keywords:** Environmental sciences, Engineering

## Abstract

This study presents the removal of phosphate from aqueous solution using a new silver nanoparticles-loaded tea activated carbon (AgNPs-TAC) material. In order to reduce costs, the tea activated carbon was produced from tea residue. Batch adsorption experiments were conducted to evaluate the effects of impregnation ratio of AgNPs and TAC, pH solution, contact time, initial phosphate concentration and dose of AgNPs-AC on removing phosphate from aqueous solution. Results show that the best conditions for phosphate adsorption occurred at the impregnation ratio AgNPs/TAC of 3% w/w, pH 3, and contact time lasting 150 min. The maximum adsorption capacity of phosphate on AgNPs-TAC determined by the Langmuir model was 13.62 mg/g at an initial phosphate concentration of 30 mg/L. The adsorption isotherm of phosphate on AgNPs-TAC fits well with both the Langmuir and Sips models. The adsorption kinetics data were also described well by the pseudo-first-order and pseudo-second-order models with high correlation coefficients of 0.978 and 0.966, respectively. The adsorption process was controlled by chemisorption through complexes and ligand exchange mechanisms. This study suggests that AgNPs-TAC is a promising, low cost adsorbent for phosphate removal from aqueous solution.

## Introduction

Phosphate is the important nutrient in the Earth’s natural ecosystem. However, large amounts of phosphate in wastewater discharged into the environment are the main reason for eutrophication in aquatic environments, resulting in serious pollution and economic problems^1^. The source of phosphate pollution usually originates from a wide range of human activities such as agricultural runoff, domestic wastewater, and industrial discharge^2^. The discharge of phosphate into aqueous ecosystems has increased in recent decades due to human activities such as industrialization. For this reason it is vital to remove phosphate from wastewater before discharging it into aquatic ecosystems. In surface water, the primary dissolved form of phosphorus is ortho phosphorus. Particulate phosphorus can exist in various forms depended on the environmental conditions. Forms of organic compounds of particulate phosphorus include algae, plant and animal tissue, waste solids, etc. The form of organic particulate phosphorus can be decomposed by microorganisms and dissolved to phosphate. Organic phosphates can enter the environment through insecticides that inhibit acetyl cholinesterase in neuromuscular systems^3^. Nutrient adsorption and allocation may be affected by sublethal concentrations of inorganic and organic phosphates. Therefore, growth of marine invertebrates is also decreased^4^.

Up till now, a number of treatment techniques including ion-exchange, biological treatment, physicochemical precipitation, membrane process, constructed wetland and adsorption methods have been used to treat phosphate contaminated wastewater^5,6^. Of these techniques, adsorption is a very promising method because of its simple operation, high efficiency and less likelihood of causing secondary pollution^7^. Several different adsorbents have been studied for phosphate adsorption from aqueous solution such as oxides and hydroxides^8^, fly ash^9^, clay minerals^10^, activated carbon^11^, and biochar^12^. Recently, nanomaterials have attracted significant scientific interest because of their exceptionally high surface area and high phosphate removal ability. In comparison to other materials, nanomaterials are very efficient in adsorbing contaminants in water^13^. In these nanomaterials, silver nanoparticles (AgNPs) have been investigated for their ability to remove pollutants in water such as cadmium^14^ and rhodamine B^15^. However, the application of silver nanoparticles in removing pollutants is still limited due to the high production costs.

Some researchers have loaded silver nanoparticles onto activated carbon to produce a new, inexpensive adsorbent for the removal of pollutants such as methylene blue^16^, malachite green^17^, and direct yellow 12^18^. These silver nanoparticles-loaded activated carbons revealed the high adsorption capacity compared with pristine activated carbon. However, to the best of our knowledge, studies on combining silver nanoparticles and activated carbon to make a new modified adsorbent for removing phosphate from aqueous solution are quite scarce. Consequently, this study develops a new adsorbent by directly loading silver nanoparticles onto activated carbon derived from tea residue (AgNPs-TAC) to remove phosphate from aqueous solution. The physico-chemical properties as well as adsorption mechanisms of phosphate on AgNPs-TAC were investigated through detailed material characterization and batch experimental results. Here, the effects of impregnation ratio (w/w) of tea activated carbon and AgNPs, pH solution, adsorption time, adsorbent dosage and initial concentrations of phosphate on adsorption capacity were investigated.

## Materials and Methods

### Materials

All chemicals, including KH_2_PO_4_, AgNO_3_, NaOH, and H_2_SO_4_ were purchased from Merck (Darmstadt, Germany). Tea residue used for activated carbon production was collected from discarded branches and leaves of tea at the Tan Cuong tea garden in Thai Nguyen City, Vietnam.

### Synthesis of silver nanoparticles

Silver nitrate (AgNO_3_) was used as the precursor to create silver nanoparticles. Silver nanoparticles (AgNPs) were prepared utilizing the hydrothermal method as described by Nguyen *et al*.^19^. Firstly, 100 mL of AgNO_3_ solution (0.001 M) was mixed with 0.2 g of starch to generate starch solutions containing Ag^+^ ions. Then the solution was stirred vigorously with a magnetic stirrer at 70 °C to ensure the mixture retained its homogeneous nature. The temperature of the solution was continuously maintained at 70 °C and 25 mL of sodium borohydrides (0.001 M) was gradually added (drop by drop) into the mixture. Finally, the resultant solution was cooled to room temperature for further usage.

### Preparation of the AgNPs-loaded tea activated carbon (AgNPs-TAC)

The tea residue was firstly washed three times with tap water, three times with distilled water and then dried in an oven at 100 °C for 24 h. The dried tea residue was then crushed and sieved to a size range from 1 to 2 cm. The ground tea residue was heated under slow pyrolysis at 400 °C for 2 h in a furnace (Nabertherm, model L3/11/B170, Germany) before being ground and sieved again to obtain a particle size of less than 0.5 mm. The new tea activated carbon (TAC) was washed, dried at 105 °C for 2 h and kept in a sealed bag before further study.

The incipient wet-impregnation technique was used to load AgNPs onto TAC as similarly described by Nguyen *et al*.^19^. The mixture of TAC and AgNPs was prepared by mixing AgNPs with TAC at various mass ratios, from 1.0–9.0% w/w in 250 mL Erlenmeyer flasks. The flasks were then shaken at 120 rpm for 24 h in the dark. After impregnation, the wet particles were filtered and dried for 2 h at 105 °C to obtain modified TAC (AgNPs-TAC). The AgNPs-TAC with an average particle size of less than 0.5 mm was used in the batch-mode adsorption experiments to remove phosphate from aqueous solution.

### Batch adsorption experiments

Initially, an adsorption experiment was conducted to identify the optimal impregnation ratio of AgNPs and TAC^16,19^. Here, 0.03 g of AgNPs-TAC produced from four impregnation ratios of 1%, 3%, 6%, and 9% was added into 50 mL Erlenmeyer flasks containing 25 mL solution of 30 mg PO_4_^3−^/L. The flasks were sealed and shaken at 120 rpm by a shaking machine (PH-2A, China) at the room temperature of 25  ±  2 °C. The liquid samples were filtered through 11 μm filters before analysing them for phosphate residual concentration. Experimental results (presented in section 3.1) show that the impregnation ratio of 3% was the best one. Thus, the AgNPs-TAC produced at this ratio was selected for detailed batch studies and material characterization.

Detailed batch experiments were conducted to evaluate the effects of various parameters^16,19,20^ (pH, initial phosphate concentration, contact time, and adsorbent dose) on the adsorption capacity of AgNPs-TAC for phosphate. Briefly, predetermined amounts of AgNPs-TAC were added into the 50 mL Erlenmeyer flasks containing 25 mL of K_2_HPO_4_ solution. The flasks were then shaken at 120 rpm by a shaking machine (PH-2A, China) at room temperature in the laboratory.

The effect of solution pH was carried out with a phosphate solution of 30 mg/L and AgNPs-TAC dose of 1.2 g/L solution. Here, the pH of the solution was adjusted from 3 to 10 by adding either H_2_SO_4_ 0.1 M or NaOH 0.1 M. Studies on the effect of contact time and adsorption kinetics were conducted at different time intervals, ranging from 5 min to 240 min at the initial phosphate concentration of 30 mg/L, adsorbent dose of 1.2 g AgNPs-TAC/L and pH solution of 8. The outcomes of initial phosphate concentrations and adsorption isotherm were found by mixing AgNPs-TAC at doses of 1.2 g/L with 25 mL phosphate concentrations ranging from 10 to 100 mg/L over a period lasting 120 min.

### Characterization of AgNPs-TAC

The variation in the surficial morphologies of TAC and AgNPs-TAC was obtained from scanning electron microscope (SEM) images of energy dispersive X-ray spectroscopy (Hitachi S-4800) equipped with EDS and SEM systems. The crystalline structures of AgNPs-TAC were examined by X-ray diffraction pattern using XRD-D8 ADVANCE, with the Cu Ka radiation (λ = 1,5417 Å). The presence of surface functional groups of AgNPs-TAC was detected using Fourier transform infrared spectroscopy (FT/IR-6300) in the 4000–500 cm^−1^ range. The pH at the point of zero charge (pH_PZC_) was determined by the shift method^21^. Determination of the surface area and the porous structure was conducted using Brunauer–Emmett–Teller (BET - BET, Builder, SSA-4300).

### Measurements

The concentration of phosphate in the samples was determined by the Vanado-molybdo phosphoric acid method with an UV–Vis spectrophotometer^22^. The adsorption capacities of phosphate onto AgNPs-TAC at time t (q_t_, mg/g) and equilibrium (q_e_, mg/g) were calculated by Eqs. (1) and (2), respectively:

1 $${q}_{e}=\frac{({C}_{o}-{C}_{e})V}{W}$$

2 $${q}_{t}=\frac{({C}_{o}-{C}_{t})V}{W}$$

where *C*_o_ (mg/L), *C*_t_ (mg/L), and *C*_e_ (mg/L) are the phosphate concentrations in solution at beginning time, any time *t*, and equilibrium, respectively; *V* (L) is the working volume of phosphate solution; and *W* (g) is the dry weight of used AgNPs-AC.

### Data analysis

All experiments were done in triplicate. All data statistics, comprising means, standard deviations, relative standard deviations and regressions (linear) were computed on SPSS software version 19.0. Wherever possible the error bars indicating the standard deviation are illustrated in the relevant figures.

## Results and Discussion

### Effect of impregnation ratio of AgNPs and TAC (AgNPs-TAC) on phosphate adsorption

To evaluate the effect of various impregnation ratios on phosphate adsorption capacity of modified TAC, the preliminary experiments were carried out with pristine TAC and TAC loaded by AgNPs at different AgNPs/TAC mass ratios (1%, 3.0%, 6.0% and 9.0%). In this study, the experiment was carried out at initial phosphate concentration of 30 mg/L, adsorption time of 60 min, adsorbent dose of 1.2 g/L at temperature of 25 ± 2 °C.

**Figure 1:**
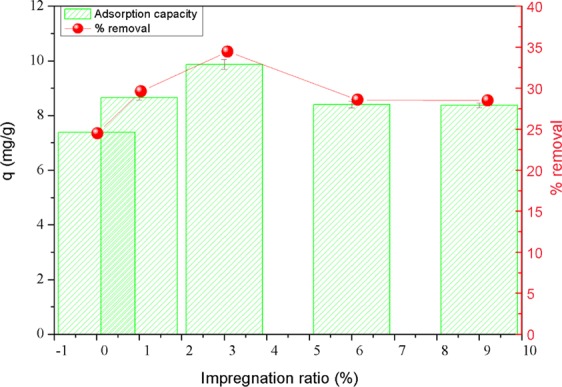
Effect of impregnation ratio of AgNPs and TAC on phosphate adsorption. Experimental conditions: initial phosphate concentration: 30 mg/L, adsorbent dose: 1.2 g AgNPs-TAC/L, pH: 3, contact time: 60 min, temperature: 25 ± 2 °C.

From Fig. 1, it can be seen that the AgNPs-TAC demonstrated a much better adsorption capacity for phosphate than the pristine TAC. The phosphate adsorption capacity increased from 7.38 mg/g to 9.87 mg/g when the impregnation ratio of AgNPs and TAC rose from 0% to 3.0%. The presence of silver nanoparticles on the AgNPs-TAC surface could trigger a higher adsorption capacity. However, the phosphate adsorption capacity fell slightly to 9.40 mg/g and 9.38 mg/g when continuously elevating the impregnation ratio of TAC in AgNPs to 6% and 9%, respectively. Furthermore the phosphate adsorption capacity did not increase when the impregnation ratio increased. This resulting trend is similar to that of a recent study on the removal of methylene blue using silver nanoparticles-loaded activated carbon derived from coconut shell^16^. According to the above finding, AgNPs-TAC with the impregnation ratio of 3% gave the highest phosphate adsorption capacity. For this reason, it was chosen for material characterization and subsequent batch experiments.

### Characterization of silver nanoparticles-loaded tea activated carbon (AgNPs-TAC)

**Figure 2:**
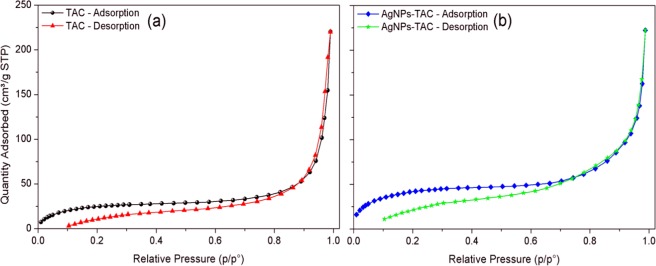
The BET results (adsorption-desorption data) of (**a**) TAC and (**b**) AgNPs-TAC.

**Figure 3:**
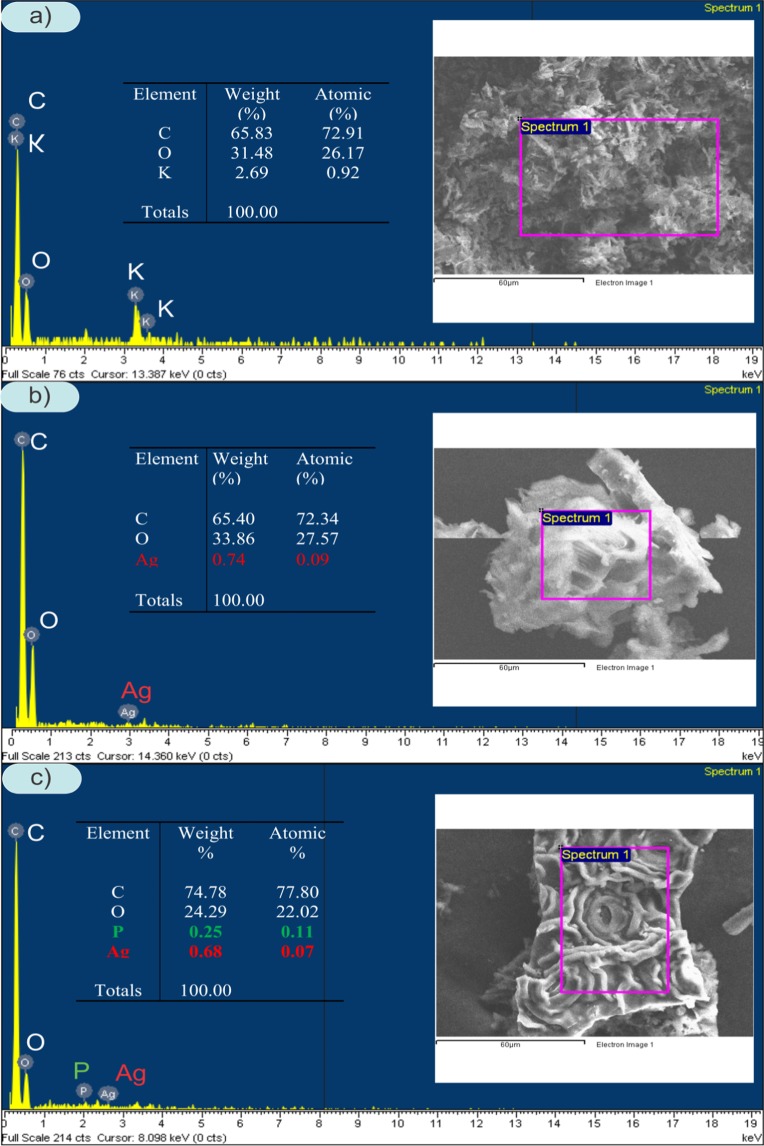
EDS spectra of TAC (**a**) and AgNPs-TAC before (**b**) and after phosphate adsorption (**c**).

**Figure 4:**
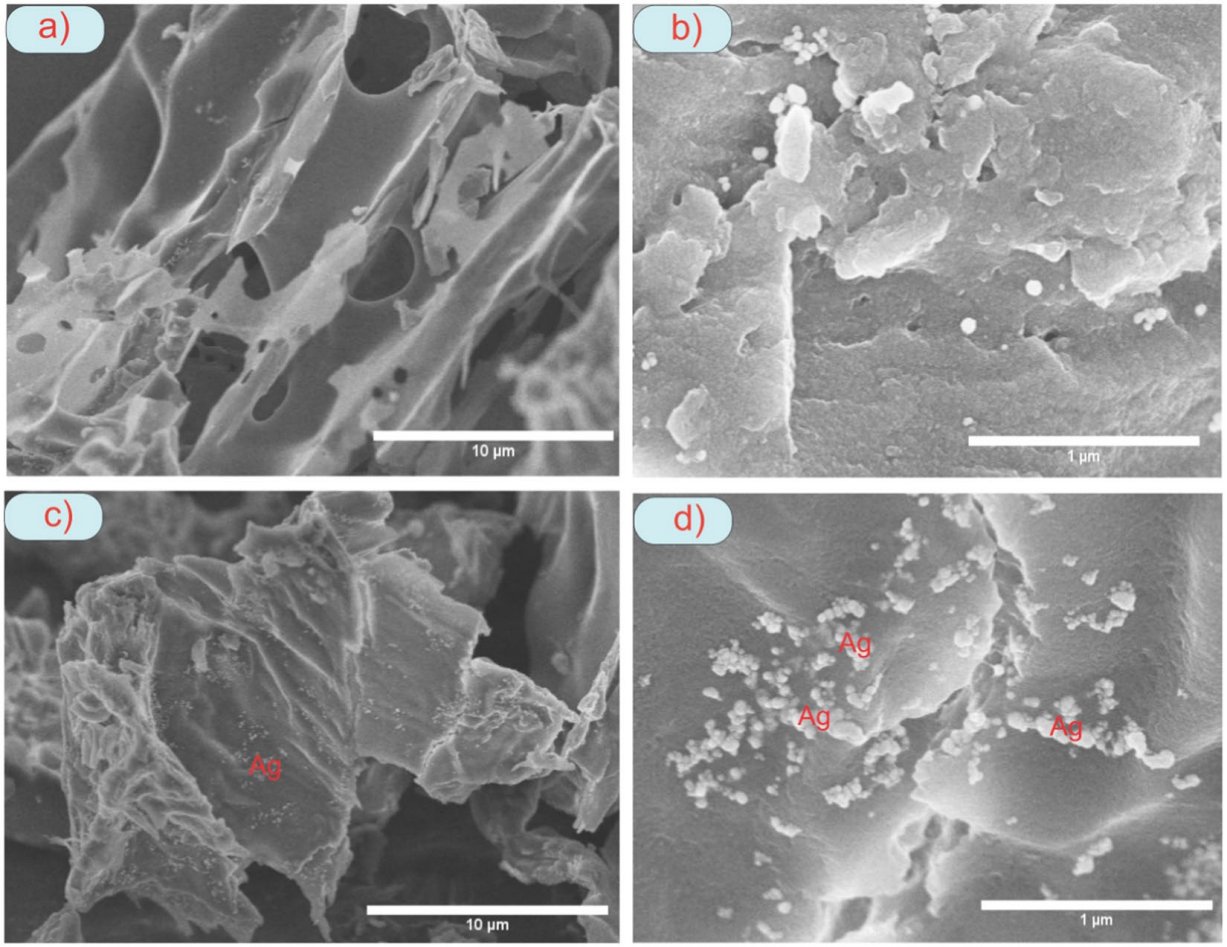
SEM image of (**a**,**b**) TAC and (**c**,**d**) AgNPs-TAC.

The surface structure is the important physical factor that affects the sorption capacity of TAC and AgNPs-TAC. The BET results (adsorption-desorption data) are presented in Fig. 2. The Brunauer–Emmett–Teller (BET) result reveals that the special surface area of TAC was 322 m^2^/g. After loading silver nanoparticles, the surface area of new AgNPs-TAC slightly increased to 349 m^2^/g. The average pore volume also rose from 0.0032 cm^3^/g to 0.0036 cm^3^/g for the TAC and AgNPs-TAC, respectively. The BET results indicated that the addition of AgNPs increased slightly the surface area and pore volume. The increase can be explained by an effective diffusion of AgNPs on the surface of TAC and the creation of new pores after coating of AgNPs on TAC^23^. The AgNPs were small particles which have higher surface area than that of TAC. The surface area and total pore volume of AgNPs-TAC may also be increased due to the nanostructure of AgNPs^24^. Increasing surface area and total pore volume of adsorbent due to modification processes confirmed the formation of new porous structures^25^. Results from EDS analysis (Fig. 3a) also indicated that the K element of TAC was released when loading AgNPs on TAC. The SEM image also showed the formation of AgNPs on the TAC (Fig. 4c,d). The surface physical morphology of AgNPs-TAC was clearly altered when white beads were present (Fig. 4c,d). These white beads did not appear in the SEM images of TAC (Fig. 4a,b), which suggests that the silver nanoparticles were distributed on the surface of AgNPs-TAC.

The elemental composition of the adsorbent before and after modification was determined by the EDS analysis. This finding indicates that the constituents of TAC included mainly C (72.91%), O (26.17%) and K (0.92%) (Fig. 3a). For AgNPs-TAC, the proportions of elements of C, O and Ag were 72.34%, 27.57% and 0.09%, respectively (Fig. 3b). This shows that silver nanoparticles were successfully attached on the TAC surface, which later affected phosphate adsorption. Moreover, the composition of AgNPs-TAC after phosphate adsorption reveals the presence of element P of 0.11% weight (Fig. 3c). It indicates that phosphate had been adsorbed on AgNPs-TAC.

**Figure 5:**
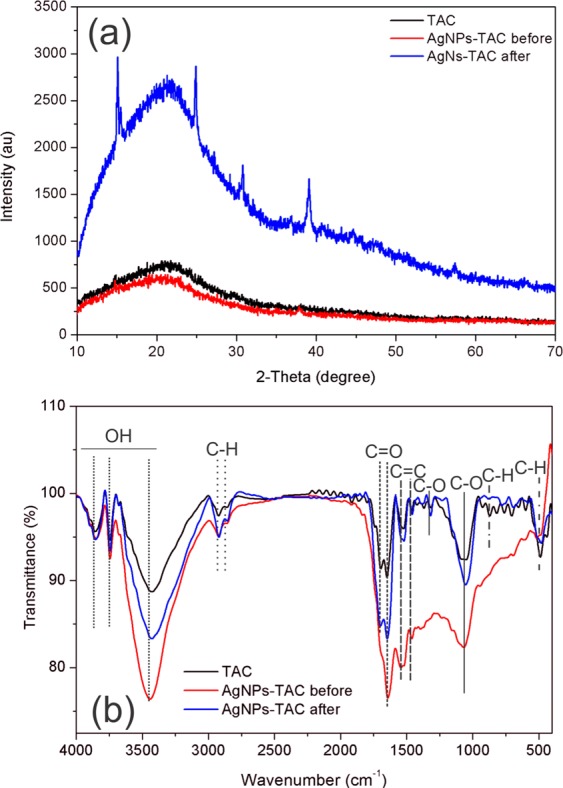
Graph of XRD (**a**) and FTIR (**b**) of TAC and AgNPs-TAC before and after phosphate adsorption.

The crystallinity of the TAC and AgNPs-TAC surface was characterized using a powder XRD analysis (Fig. 5a). The graph indicates that both TAC and AgNPs-TAC before and after phosphate adsorption included graphite crystal structure of diffraction peaks at 21.33^o^. This result also shows that after impregnation with AgNPs, the AgNPs-TAC’s surface appeared to have silver nanoparticles at peaks of 37.88^o^ and 44.01^o^ (Fig. 5a). This proved to be similar to recent reports in which silver nanoparticles were loaded ont activated carbon at the peaks of 37.9^o^ ^26^ and 44^o^ ^27^.

The dominant functional groups on the TAC, AgNPs-TAC before and after phosphate adsorption were present as shown by FTIR spectra in Fig. 5b. From Fig. 5b, it can be seen that the broad peaks of 3851 cm^−1^, 3739 cm^−1^, and 3434 cm^−1^ were assigned to the OH stretching, which indicated compounds of phenols, alcohols and absorbed water^28,29^. The peaks at around 2922 cm^−1^ and 2855 cm^−1^ corresponded to the C-H stretching of alkanes and alkenes^29^. The C=O stretching vibration in carbonyls appeared at peaks of 1698 cm^−1^ and 1692 cm^−1^, which confirmed the presence of carboxylic acids, lactone and carbonyl groups^16,28^. The peaks at 1531 cm^−1^ and 1458 cm^−1^ indicated the C=C group of aromatic rings^30^.

The presence of the C-O stretching group in acids, alcohols, phenols, ethers and esters can be recognized at peaks of 1320 cm^−1^ and 1058 cm^−1^ ^28^. The peaks from 597 cm^−1^ to 869 cm^−1^ contributed to the C-H out-of-plane bending in benzene derivatives^31^. In AgNPs-TAC, the peaks at the 3434 cm^−1^ − 3851 cm^−1^, around 2922 cm^−1^, 1531 cm^−1^ and 1058 cm^−1^ range became stronger than that in TAC. This reveals that the presence of Ag^+^ ions affected functional groups on the AgNPs-TAC’s surface. The point of zero charge (pH_PZC_) of TAC was 6.15 and pH_PZC_ of AgNPs-TAC before and after phosphate adsorption were 6.52 and 6.58, respectively. Results indicate that pH_PZC_ of AgNPs-TAC did not significantly change after the adsorption process.

### Effect of pH

**Figure 6:**
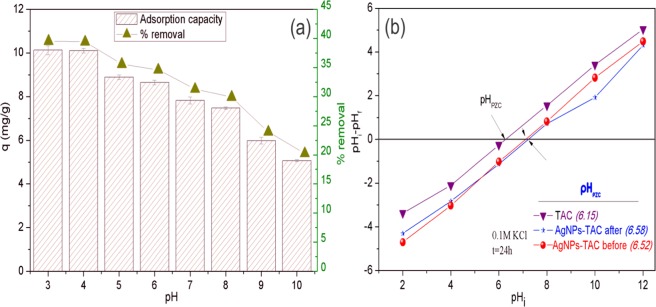
(**a**) Effect of pH on phosphate adsorption. Experimental conditions: initial phosphate concentration: 30 mg/L, adsorbent dose: 1.2 g AgNPs-TAC/L, contact time: 60 min, temperature: 25 ± 2 °C and (**b**) pH_PZC_ of AgNPs-TAC.

The pH of the solution determines the presence of phosphate hydroxyl groups such as H_3_PO_4_, H_2_PO_4_^−^, HPO_4_^2−^ and PO_4_^3−^. This is one of the most important parameters influencing the adsorption process of phosphate^5,32^. The effect of the solution pH on the phosphate adsorption of AgNPs-TAC was examined within a pH range of 3.0 to 10.0 and the results are shown in Fig. 6. It could be observed that the adsorption strongly depended on pH. From Fig. 6, as can be seen that the adsorption capacity of phosphate onto AgNPs-TAC decreased from 10.13 to 5.06 and the removal efficiency declined from 39.53% to 20.26% when pH increased from 3 to 10. The adsorption capacity of phosphate decreased significantly when solution pH was more than 6. A similar result on phosphate removal by Zr/Al-pillared montmorillonite was reported by Fang *et al*.^33^.

At different pH values, the phosphate can exist in different forms of H_2_PO_4_^−^, HPO_4_^2−^, and PO_4_^3−^ ^34^. In acidic conditions, the main phosphate species are monovalent H_2_PO_4_^−^ and HPO_4_^2−^ ^11^. However, in alkaline conditions, the phosphate exists in the form of PO_4_^3−^. Moreover, the pH_pzc_ values of the AgNPs-TAC were 6.52 and 6.58 before and after phosphate adsorption process, respectively. When solution pH was higher than the pH_pzc_ value of the AgNPs-TAC, the surface of AgNPs-TAC was positively charged. However, the phosphate adsorption capability of AgNPs-TAC decreased with the increase in solution pH. It suggests that electrostatic attraction was not the main phosphate removal mechanism. The competition of OH^−^ with PO_4_^3−^ in alkaline conditions could also contribute to a loss of phosphate adsorption onto AgNPs-TAC when increasing solution pH.

### Effect of contact time

**Figure 7:**
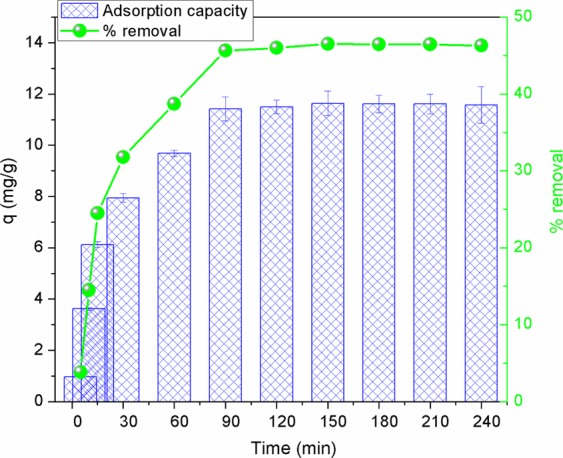
Effect of contact time on phosphate adsorption onto AgNPs-TAC. Experimental conditions: initial phosphate concentration: 30 mg/L, adsorbent dose: 1.2 g AgNPs-TAC/L, pH: 3, temperature: 25 ± 2 °C.

Adsorption experiments were carried out at different contact times from 5 to 240 min with the initial phosphate concentration of 30 mg/L, adsorbent dose of 1.2 mg/L at solution pH of 3 and room temperature (25 ± 2 °C). Results from Fig. 7 show that the adsorption capacity of phosphate increased rapidly, from 0.96 mg/g to 11.50 mg/g when increasing contact time from 30 to 120 min (corresponding to phosphate removal rate rose from 3.8% to 46%). In this experimental scenario, the adsorption process slowed down after 120 min and became stable after 150 min. Adsorption capacity and the efficiency in removing phosphate were achieved the highest values of 11.63 mg/g and 46.53%, respectively, at 150 min contact time. In the initial stage, the AgNPs-TAC’ surface had a large number of fresh available binding sites leading to a rapid increase in adsorption^6^. Over a period of time, the adsorption process become slower and stable due to: firstly, the saturation of adsorption active sites on the surface of AgNPs-TAC; and secondly, gradual reduction in the concentration gradient between the bulk solution and adsorbent^1^. This trend was similar to results for other studies that looked at phosphate adsorption from aqueous solution^34,35^.

### Effect of absorbent dose

**Figure 8:**
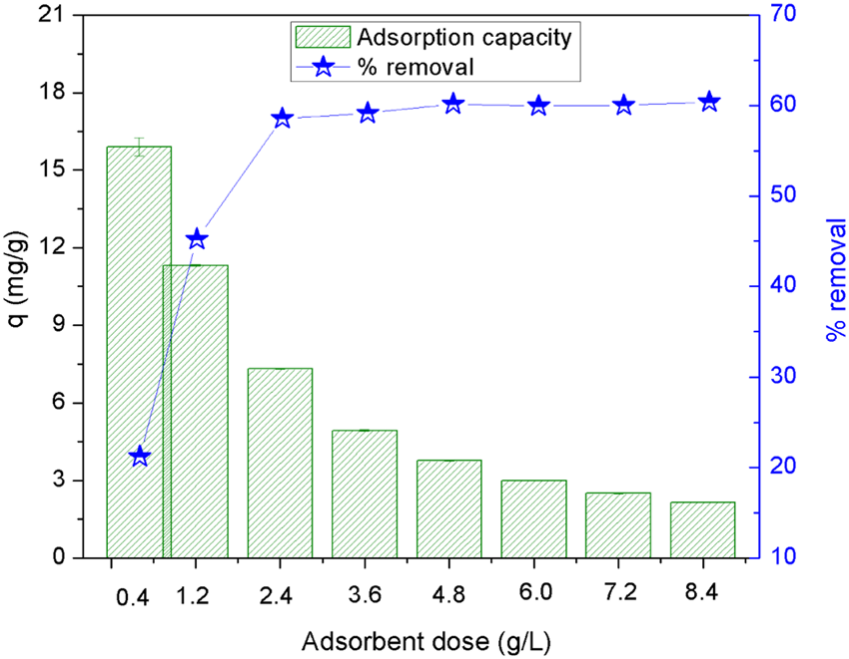
Effect of AgNPs-TAC dosage on phosphate adsorption. Experimental conditions: pH: 3, contact time: 150 min, initial concentration of PO_4_^3−^: 30 mg/L, temperature: 25 ± 2 °C.

To examine the possible effects of adsorbent dose on the phosphate adsorption of AgNPs-TAC, experiments were conducted at different dosages ranging from 0.4 to 8.4 g/L. Experiments were carried out at the optimum pH of 3.0, contact time lasting 120 min and initial phosphate concentration of 30 mg/L. The results are shown in Fig. 8. It could be observed that the adsorption percentage of phosphate increased from 21.2% to 60.2%, which corresponded to the AgNPs-TAC dose ranging from 0.4 to 4.8 g/L. Its increase could be caused by the increase in the available surface area and active sites for adsorption when raising the dose of AgNPs-TAC^6^. However, the removal efficiency of phosphate did not increase and remained stable when the dose rose from 4.8 to 8.4 g/L. Moreover, the adsorption capacity of phosphate onto AgNPs-TAC tended to decrease from 15.90 mg/g to 2.15 mg/g when the AgNPs-TAC dosage rose from 0.4 to 8.4 g/L. The decline in adsorption capacity at a higher adsorbent dose might be due to aggregation of AgNPs-TAC particles which reduced the effective surface area and active sites for phosphate adsorption^36^. Here, the aggregation at higher adsorbent dose of AgNPs-TAC at a certain initial phosphate concentration and solution volume led to an increase in the unsaturation of sorption sites on AgNPs-TAC surface. It triggered a decrease in adsorption capacity^37^. These results are in line with other studies which indicated that adsorbent dose plays a vital role in the adsorption process^38,39^.

### Effect of initial phosphate concentration

**Figure 9:**
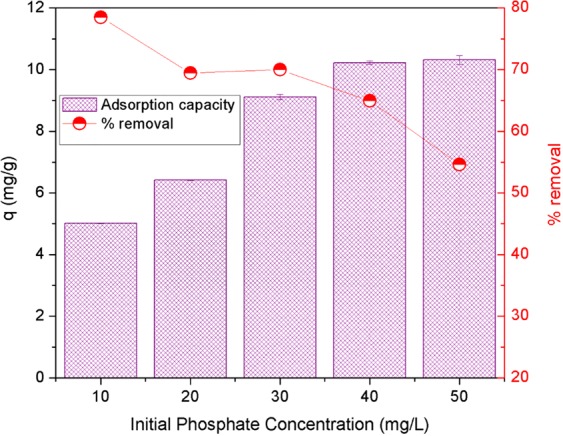
Effect of initial phosphate concentrations on the adsorption capacity by AgNPs-TAC. Experimental conditions: solution pH: 3, contact time: 150 min, adsorbent dose: 1.2 g AgNPs-TAC/L solution, temperature: 25 ± 2 °C.

The initial concentration of adsorbate extremely influenced the adsorption process. The effect of initial phosphate concentration was investigated at different initial phosphate concentrations from 10 to 50 mg/L at optimum pH of 3, adsorbent dosage of 1.2 g/L and contact time of 120 min. Results (Fig. 9) show that the adsorption capacity of phosphate on AgNPs-TAC increased significantly as the initial phosphate concentration rose from 10 mg/L to 40 mg/L and became stable at a higher concentration. The adsorption capacity reached a maximum of 10.23 mg/g at the initial phosphate concentration of 40 mg/g. Meanwhile, the removal efficiency of phosphate fell from 78.50% to 54.62%, which corresponded with an increase in the initial phosphate concentration from 10 mg/L to 50 mg/L. This is due to the fact that at a lower concentration, almost all phosphate ions interacted with available binding sites. At a higher initial phosphate concentration, the binding sites became saturated and adsorption capacity did not rise further at the higher initial phosphate concentration^6^. Furthermore, when the adsorbent mass and volume of solution were unchanged, the ratio of active sites to the phosphate ions concentration became lower at a higher initial concentration. This led to a decrease in the percentage of phosphate removed^36^. Similar results have been reported elsewhere^6,28^.

### Adsorption isotherm

To further understand the adsorption of phosphate on AgNPs-TAC, three isotherm models, namely the Langmuir, Freundlich and Sips were employed to describe the adsorption isotherm. The Langmuir model presumes that a monolayer adsorption process takes place on a homogenous surface and adsorption energy of all active sites is always similar^40^. The Freundlich model assumes that multilayer adsorption occurs on a heterogeneous surface and all the adsorption locations have different affinities^6^. The Sips model is a combination of the Freundlich and Langmuir models^41^. The equations for the Langmuir, Freundlich and Sips models are shown below in Eqs. (3), (4) and (5), respectively:

3 $${q}_{e}=\frac{{q}_{m}{K}_{L}{C}_{e}}{1+{K}_{L}{C}_{e}}$$

4 $${q}_{e}={K}_{F}{C}_{e}^{\frac{1}{n}}$$

5 $${q}_{e}=\frac{{q}_{m}{(b{C}_{e})}^{\frac{1}{n}}}{1+{(b{C}_{e})}^{\frac{1}{n}}}$$

where *q*_*e*_ (mg/g) and *q*_*m*_ (mg/g) are the adsorption capacity at equilibrium and the maximum saturated adsorption capacity; *C*_*e*_ (mg/L) is the adsorbate concentration at equilibrium; *K*_*L*_ (L/mg) is the Langmuir constant related to the energy of the adsorption; and *K*_*F*_ (mg/g) is the Freundlich constant, which characterizes the strength of adsorption.

**Figure 10:**
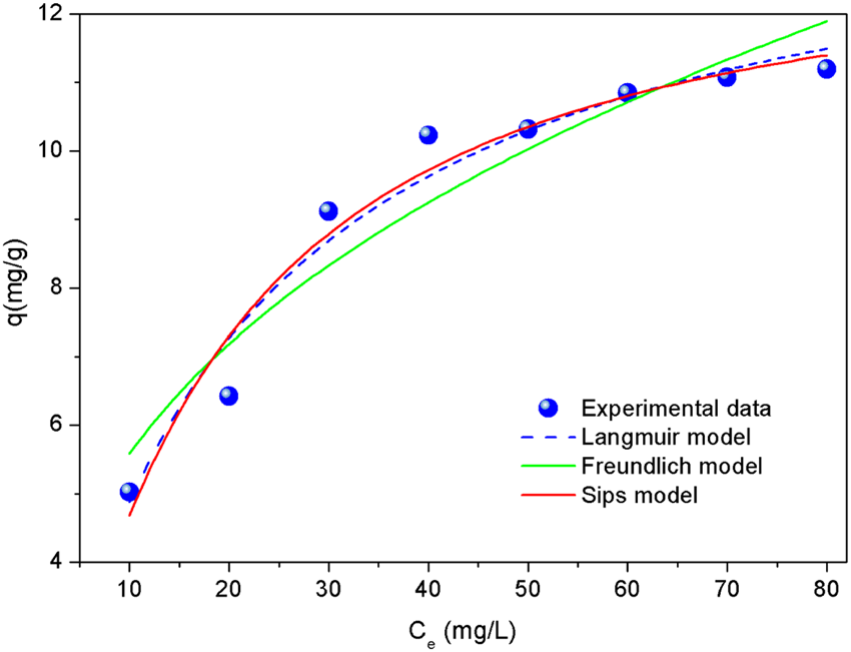
Adsorption isotherm of phosphate on AgNPs-TAC at contact time of 120 min, AgNPs-TAC dose of 1.2 g/L, solution pH: 3 and adsorbent dose: 1.2 g AgNPs-TAC/L.

The fitting results for the experimental data of phosphate adsorption on AgNPs-TAC by Langmuir, Freundlich and Sips models are shown in Fig. 10 and isotherm parameters are presented in Table 1. It can be observed that the adsorption data fitted well to the Langmuir, Freundlich and Sips models with the interrelated coefficients values (*R*^2^) of 0.957, 0.902 and 0.951, respectively. It indicates that the Langmuir and Sips models did fit better than the Freundlich model for isotherm adsorption of phosphate on AgNPs-TAC. Q_m_ values determined by the Langmuir and Sips model were 13.62 mg/g and 13.22 mg/g, respectively, which were closer to the experimental value (q_mexp_ 11.19 mg/g). It indicates that the main adsorption occurred on the monolayer or through a fixed number of identical sites on the AgNPs-TAC surface. Here, the 1/n value calculated from the Freundlich model was 0.306. It indicates that the phosphate adsorption onto AgNPs-TAC was favorable. A similar trend was reported for the removal of Cr(VI) by magnetic biochar^42^ and magnetite nanoparticles^43^.

**Table 1:** Adsorption isotherm parameters and correlation coefficients of the Langmuir, Freundlich and Sips models for phosphate adsorption on AgNPs-TAC.

Langmuir model			Freundlich model			Sips model				q_mexp_ (mg/g)
*q*_*m*_ (mg/g)	*K*_*L*_	*R*^2^	*K*_*F*_	*1/n*	*R*^2^	*q*_*m*_(mg/g)	*1/n*	*b*	*R*^2^	
13.62	0.052	0.957	0.916	0.363	0.902	13.22	1.172	0.0369	0.951	11.19

### Adsorption kinetics of phosphate on AgNPs-TAC

In order to describe the kinetics of phosphate adsorption on AgNPs-TAC, two kinetics models including pseudo-first-order (Eq. 6) and pseudo-second-order (Eq. 7) models were used.

6 $${q}_{t}={q}_{e}(1-{{\rm{e}}}^{-{k}_{1}t})$$

7 $${q}_{t}=\frac{{q}_{e}^{2}{k}_{2}t}{1+{q}_{e}{k}_{2}t}$$

Where, q_e_ and q_t_ are the adsorption capacity at equilibrium and at time t (mg/g); k_1_ is the first-order rate constant (min^−1^); k_2_ is the second-order rate constant, g/mg.min; α is the initial adsorption rate (mg/g min); and β is the adsorption constant (g/mg).

**Figure 11:**
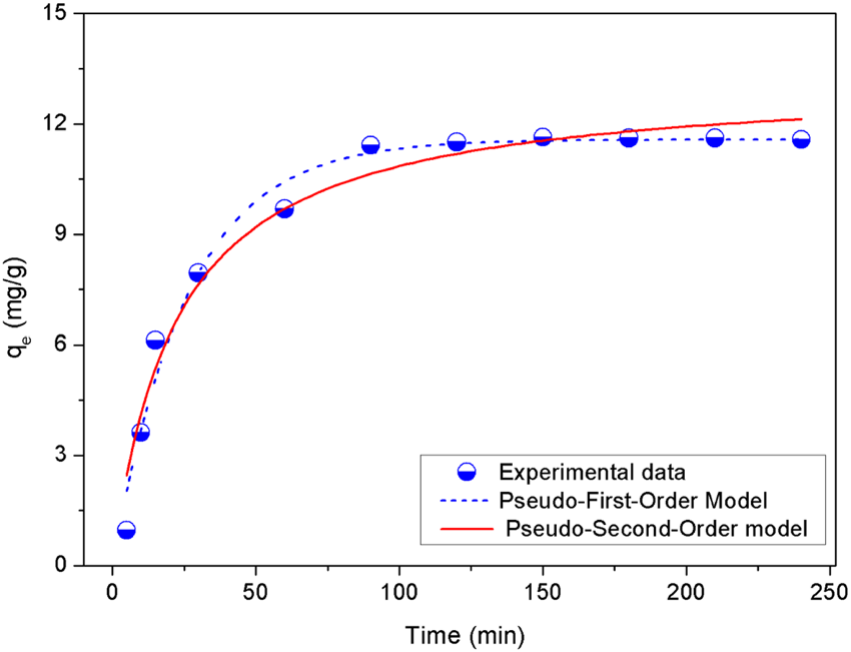
Kinetics models of phosphate adsorption on AgNPs-TAC with initial phosphate concentration of 30 mg/L, adsorbent dose of 1.2 g/L, solution pH: 3 and contact time of 120 min.

The corresponding kinetics parameters and prediction curves of the two kinetics models are presented in Table 2 and Fig. 11, respectively. The high values of linear regression coefficient (R^2^ from 0.978 to 0.966) indicate the phosphate adsorption onto AgNPs-TAC fit well to both models. Correlation coefficient of the pseudo-first-order model (R^2^ = 0.978) was slightly higher than that of the pseudo-second-order model (R^2^ = 0.966). Additionally, the maximum adsorption capacity (q_m_) calculated from the pseudo-first-order model (11.57 mg/g) and the pseudo-second-order model (13.24 mg/g) were relatively close to the experimental data (11.63 mg/g). These results indicated that both pseudo-first-order and pseudo-second-order models could describe the adsorption kinetics of phosphate on AgNPs-TAC. They also illustrated that chemical sorption was the main mechanism for phosphate adsorption on AgNPs-TAC which involves valence forces through sharing or exchange of electrons between the adsorbate and adsorbent^2^. These results are similar to what is documented in previous studies on phosphate adsorption using other adsorbents^6,33^.

**Table 2:** Kinetics parameters for phosphate adsorption on AgNPs-TAC.

Pseudo-first order			Pseudo-second order			q_e,exp_ (mg/g)
q_m,cal_ (mg/g)	K_1_ (1/min)	R^2^	q_m,cal_ (mg/g)	K_2_ (g/mg × min)	R^2^	
11.57	0.0386	0.978	13.24	0.0034	0.966	11.63

### Discussion of AgNPs-TAC role in phosphate adsorption

The adsorption mechanisms of phosphate by activated carbon were affected by the surface properties of AgNPs-TAC. The main operating mechanism of phosphate by AgNPs-TAC was the ion exchange process. To identify phosphate adsorption mechanisms on TAC and AgNPs-TAC samples were characterized by BET, SEM, XRD, FTIR and EDS. Results of the BET analysis show that the AgNPs-TAC led to a negligible increase in surface area (348 m^2^/g) and pore volume (0.0036 cm^3^/g) than TAC (321.75 m^2^/g and 0.0032 cm^3^/g, respectively). The increase of surface area and number of active sites due to the coating of Ag nanoparticles on activated carbon (AgNPs–TAC) led to the increase in phosphate adsorption on AgNPs-TAC. The XRD analysis also revealed that the AgNPs loaded successful on the TAC surface. The presence of Ag^+^ ions affected functional groups located on the AgNPs-TAC’s surface, and this was further confirmed through the bigger peaks of AgNPs–TAC in the following ranges: 3434 cm^−1^ to 3851 cm^−1^, 2922 cm^−1^, 1531 cm^−1^ and 1058 cm^−1^.

The good fit of the experimental data to the adsorption kinetics and isotherm models also confirmed that the removal of phosphate by AgNPs-TAC occurred through chemisorption. The adsorption process resulted in the formation of coulombic attraction between phosphate anions and the binding sites of AgNPs-TAC. This was proven by the presence of P on the surface of AgNPs-TAC (Fig. 3c). At pH of 3, the phosphate exist in the form of dihydrogen phosphate ions (H_2_PO_4_^−^) and the surface complex is protonated. Thus, the possible complexes were Ag(PO)(OH), Ag_2_P(OH)_2_, and/or Ag_2_(PO)(OH)_2_. The form of H_2_PO_4_^−^ could also combine with Ag^+^ to form Ag_2_P precipitate. It is observed that the peak of silver nanoparticles was changed from weak at 37.88^o^ to stronger at 39.13^o^ for AgNPs-TAC before and after phosphate adsorption process. This may be due to the possible complexes of silver nanoparticles and phosphate. Moreover, at low pH surface hydroxyl groups of AgNPs-TAC were protonated in the ligand exchange process and -OH^2+^ was to displace easily from the metal binding sites^44^. Thus, ligand exchange was also one of mechanisms of phosphate adsorption onto AgNPs-TAC. For AgNPs-TAC, after phosphate adsorption, two peaks at 1698 cm^−1^, 1320 cm^−1^, and some peaks around 597 cm^−1^ to 869 cm^−1^ were virtually disappeared. This obviously indicates that some surface groups of the adsorbent were replaced by phosphate. Therefore, the complexes and ligand exchange were the main mechanisms of the phosphate adsorption onto AgNPs-TAC. This agreed with the previous report that anionic coordination exchange adsorption was the main mechanism of phosphate adsorption onto modified bentonite granular (MBG)^45^.

To assess phosphate adsorption, the results documented in this study were compared with other adsorbents. The findings for phosphate adsorption with various other types of adsorbents noted in seven studies are presented in Table 3. The data in Table 3 showed that phosphate adsorption capacity of AgNPs-TAC was higher than that of fly ash (FA)/biochar composite, Lanthanum doped vesuvianite, activated laterite, Palygorskite with acid and thermal treatment and red mud with HCl acid treatment. However, the adsorption capacity of AgNPs-TAC for phosphate was lower than that of Zr/Al-pillared montmorillonite, carboxymethyl konjac glucomannan loaded with lanthanum. This indicated that AgNPs-TAC is a potentially effective and low-cost adsorbent for removing phosphate from aqueous solutions.

**Table 3:** Comparison of AgNPs-TAC with other adsorbents for phosphate adsorption capacity.

Adsorbsent	Equilibrium phosphate concentration range (mg/L)	Langmuir maximum adsorption capacity (mg/g)	References
AgNPs-TAC	0–50	13.62	This study
Fly ash (FA)/biochar composite	0–25	3.20	^26^
Carboxymethyl konjac glucomannan loaded with lanthanum	0–16	17.156	^4^
Zr/Al-pillared montmorillonite	0–15	17.2	^28^
Activated laterite	0–25	1.86	^37^
Lanthanum doped vesuvianite	0–4	6.7	^38^
Red mud with HCl acid treatment	0–1	0.58	^39^
Palygorskite with acid and thermal treatment	0–150	8.31	^40^

### Reusability

**Figure 12:**
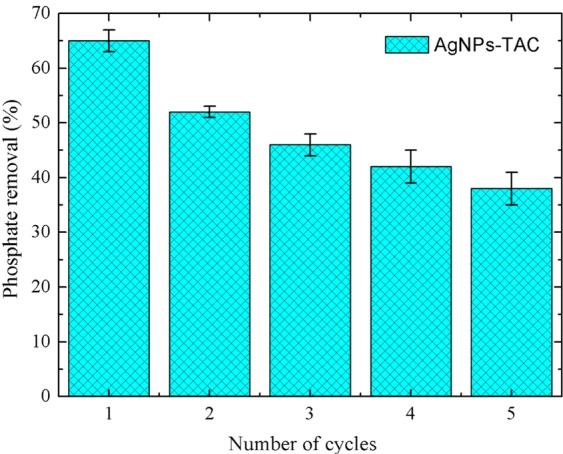
Phosphate percentage removal of AgNPs-TAC after regeneration by NaOH 1 M. Initial phosphate concentration of 30 mg/L, adsorbent dose of 1.2 g/L, solution pH: 3 and contact time of 120 min.

The reusability of AgNPs-TAC was evaluated by regeneration of P-loaded AgNPs-TAC after adsorption. AgNPs-TAC was firstly separated by filtration after the batch adsorption experiment. The active sites on the surface of AgNPs-TAC were then regenerated by mixing 0.1 g of P-loaded AgNPs-TAC with 25 mL NaOH 1 M during 24 h. Finally, samples were dried at 80 °C under vacuum condition before reusing in the batch study. Figure 12 presents the result of the reusability of AgNPs-TAC for phosphate adsorption. From Fig. 12, phosphate adsorption efficiency onto AgNPs-TAC decreased gradually in the consecutive five regeneration cycles, from 65.39% (first adsorption) to 38.26% (after five cycles). The decrease of adsorption efficiency could be caused by the loss of functional groups and incomplete desorption^46^. This result indicates that AgNPs-TAC can be reusable for phosphate removal from water and wastewater within limited times.

## Conclusions

AgNPs-TAC was composited for the first time as an adsorbent for phosphate removal from aqueous solution. The phosphate adsorption process on AgNPs-TAC greatly depended on pH value. In this study, the pH of 3 was the best one for the adsorption of phosphate on AgNPs-AC. This result indicates that adsorption capacity of phosphate on AgNPs-AC increased when the AgNPs-TAC dose and contact time also increased. The maximum adsorption capacity of phosphate by AgNPs-TAC was 13.62 mg/g at an initial phosphate concentration of 30 mg/L. As well, the Langmuir and Sips models were suitable models for describing the adsorption isotherm of phosphate on AgNPs-TAC. The pseudo-first-order and pseudo-second-order models had the best fit for the adsorption kinetics of phosphate on AgNPs-TAC with high correlation coefficients of 0.978 and 0.966, respectively. Finally, the main mechanism of phosphate adsorption onto AgNPs-TAC was ion exchange, evidenced by the sharing or exchange of electrons between the adsorbate and adsorbent.
